# Interpreting Intra-site Spatial Patterns in Seasonal Contexts: an Ethnoarchaeological Case Study from the Western Alps

**DOI:** 10.1007/s10816-015-9268-5

**Published:** 2015-12-07

**Authors:** Francesco Carrer

**Affiliations:** 1 Department of Archaeology, University of York, King’s Manor, York, YO1 7EP UK; 2 McCord Centre for Historic and Cultural Landscapes, Newcastle University, Armstrong Building, Queen Victoria Road, Newcastle upon Tyne, NE1 7RU UK

**Keywords:** Intra-site patterns, Ethnoarchaeology, Spatial analysis, Western Alps, Activity areas, Trampling

## Abstract

This paper deals with the ethnoarchaeological analysis of the spatial pattern of artefacts and ecofacts within two traditional pastoral huts (a dwelling and a seasonal dairy) in the uplands of Val Maudagna (Cuneo province, Italian western Alps). The composition of the ethnoarchaeological assemblages of the two huts was studied and compared; point pattern analysis was applied to identify spatial processes mirrored in the interactions between objects; Moran’s *I* correlogram and empirical variogram were used to investigate the effects of trampling on the displacement of objects on the floor. The results were compared with information provided by the herder who still used the huts. The quantitative and ethnographical data enabled inferences to be made that can help in the interpretation of archaeological seasonal sites. The function of a seasonal site can be recognized, as can the impact of delayed curation on the composition of the assemblage and the importance of the intensity of occupation compared with the frequency of occupation. The spatial organization of activities is reflected in the spatial patterns of objects, with clearer identification of activity areas in intensively occupied sites, and there is evidence for the behaviour behind the spatial segregation of activities. Trampling is a crucial post-depositional factor in the displacement of artefacts and ecofacts, especially in non-intensively exploited sites. From a methodological point of view, this research is another example that highlights the importance of integrating quantitative methods (especially spatial analysis and geostatistical methods) and ethnoarchaeological data in order to improve the interpretation of archaeological sites and assemblages.

**Electronic supplementary material:**

The online version of this article (doi:10.1007/s10816-015-9268-5) contains supplementary material, which is available to authorized users.

## Introduction

The main aim of studies of spatial patterning within archaeological sites is the identification of activity areas in order to reconstruct and explain past behaviours and social relationships (Carr 1984). Since the 1970s, ethnoarchaeology has provided fundamental interpretative tools for addressing the relationships between use of space and spatial patterns (David and Kramer 2001, pp. 255–283). Important early projects were carried out among hunter-gatherers (Binford 1978; O’Connell 1987; Yellen 1977) and pastoral communities (Cribb 1991; Gifford 1978; Simms 1988) and tried to correlate spatial behaviour with the dispersion/clustering of archaeological assemblages. Most of these studies suggested that specific intra-site activity areas were impossible to identify, not just because of the limited visibility of mobile groups within the archaeological record, but also because post-depositional processes were assumed to hide the original spatial organization. These early studies all recognized the role of trampling in the displacement of objects from their primary location, but none of them focused specifically on it. It was experimental archaeology that provided data that enabled the actual impact of trampling in the spatial organization and dimensional composition of archaeological assemblages to be inferred (Domínguez-Rodrigo *et al*. 2009; Gifford-Gonzalez *et al.* 1985; McBrearty *et al*. 1998; Nielsen 1991; Olsen and Shipman 1988; Shea and Klenck 1993; Stockton 1973; Villa and Courtin 1983).

Although these studies were important to enhance our understanding of the complex processes that occur in the formation of the archaeological record (see Schiffer 1987). the applicability of their inferences for the analysis of archaeological spatial patterns was often quite limited. The observed ethnoarchaeological or experimental contexts often returned biased and idiosyncratic patterns that were difficult to compare with archaeological ones. This problem can be avoided using state-of-the-art quantitative methods, which enable generalizable and reliable analogical models to be created and tested in archaeological case-studies (Carr 1984, pp. 104–105). In this paper, spatial statistics and geostatistics were applied to investigate assemblage spatial patterns within two modern pastoral huts of the Val Maudagna (Cuneo province, Piemonte, Italy). The aim was to assess whether these quantitative methods could be used to identify intra-site activity areas and post-depositional processes in ethnoarchaeological sites and whether this identification could be useful for archaeological interpretation.

The relationship between the assemblage composition and size and site exploitation, the recognizability of activity areas and the effects of trampling were each addressed. Particular attention was also given to the effect of seasonality on assemblage composition.

## Pastoralism and Pastoral Structures in the Western Alps

**Fig. 1:**
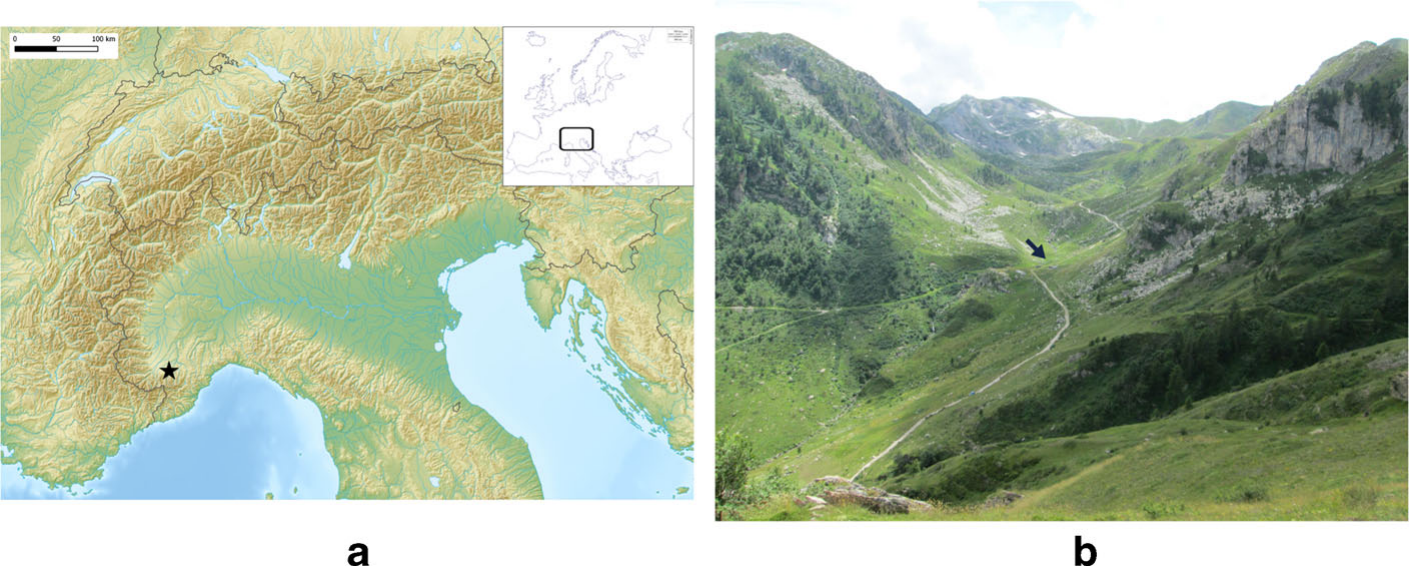
The location of Monregalese (*star*) within the Alps (**a**) and of *gias della Brignola* (*arrow*) within Val della Brignola (**b**)

Seasonal pastoralism is still an important economic strategy in the Italian western Alps. In the Ligurian and Maritimes Alps, local herders (*margari*) take their livestock to the uplands during the summer. There, they exploit traditional seasonal sites called *gias* (Casanova 2002). which are composed of four functional units: a dwelling for the herder(s), an open space for stabling the livestock, a milk-processing/cheese-making structure and a structure for maturing the cheese. The number, shape and characteristics of these units vary from area to area. In the Monregalese (Val Casotto, Val Corsaglia, Val Maudagna and Val Ellero), part of the Cuneo province (Fig. 1a), each *gias* is composed of two twin huts (called *casót*) and a cellar (called *sella*) for maturing the cheese (the *sella* is shared by different nearby *gias*). The twin huts have two different functions: one (the *casót del fogo*) is used as a temporary dwelling by the herder, the other (the *casót del latte*) is used as a seasonal dairy. The twin huts have more or less the same dimensions (on average an internal surface of 10–15 m^2^), are partially underground and are composed of a low dry-stone wall and a wooden-pole roof covered with hay or turf (or today with metal sheets and nylon fabric) (Mamino 2001; Roatta and Roatta 1999). The cellar is almost totally underground, with dry-stone or concrete walls and a stone-slab roof covered with turf. The huts were traditionally built (or re-built) by the herders every summer, while the cellar was provided and restored by the local municipality. Nowadays, several of the traditional huts have been abandoned and replaced by new and more comfortable refuges provided by the municipalities; most of the traditional cellars, however, are still exploited as cheese cellars.

Three different *gias* are exploited by the herders during different periods of the summer, according to the progressive growth of the grass. A lower *gias* (the *gias sottano*) is occupied for (up to) 15 days at the beginning of the summer (late June) and for (up to) 15 days at the end of the summer (early September); an intermediate *gias* (the *secondo gias*) and a higher *gias* (the *gias soprano*) are occupied for 20–25 days in July and August, respectively (Rosso 1950, p. 23–24). This strategy avoids over-grazing and over-manuring of the pastures and stabling areas around the *gias*.

Dairy products are the most important outcomes of seasonal pastoralism in the area (Rosso 1950). Raschera, a hard cheese of cattle, goat and sheep milk, is the most famous and valuable dairying product in these valleys (Cevasco and Poggi 1999). However, recent health and safety regulations from the European Union (EU) have forbidden the production of cheese within the traditional dry-stone upland huts. This has increased the abandonment of these structures and encouraged the construction of modern seasonal huts and the establishment of cooperative dairies in the lowlands.

## Case Study: The *gias della Brignola* (FB018A-B)

The study of the *gias della Brignola* is part of a wider ethnoarchaeological project (EthWAL) that began in the summer of 2013. During the preliminary phases of the research, several pastoral sites were recorded at higher elevations of Val Maudagna, and an alphanumeric identification was given to each. Val della Brignola (municipality of Magliano Alpi), a secondary upland valley of Val Maudagna (Fig. 1b), was selected as the sample area for this study because it is one of the last areas of the Cuneo province where traditional *gias* are still exploited (perhaps because of the lack of modern seasonal huts).

A local herder used to rent the pastures of Val della Brignola (with their *gias*) from the municipality and graze a herd of cattle (mainly Piemontese cattle) there. He used to produce Raschera cheese during the summer but the EU regulations forced him to stop a few years ago. He still produces fresh cheese for personal consumption from time to time.

**Fig. 2:**
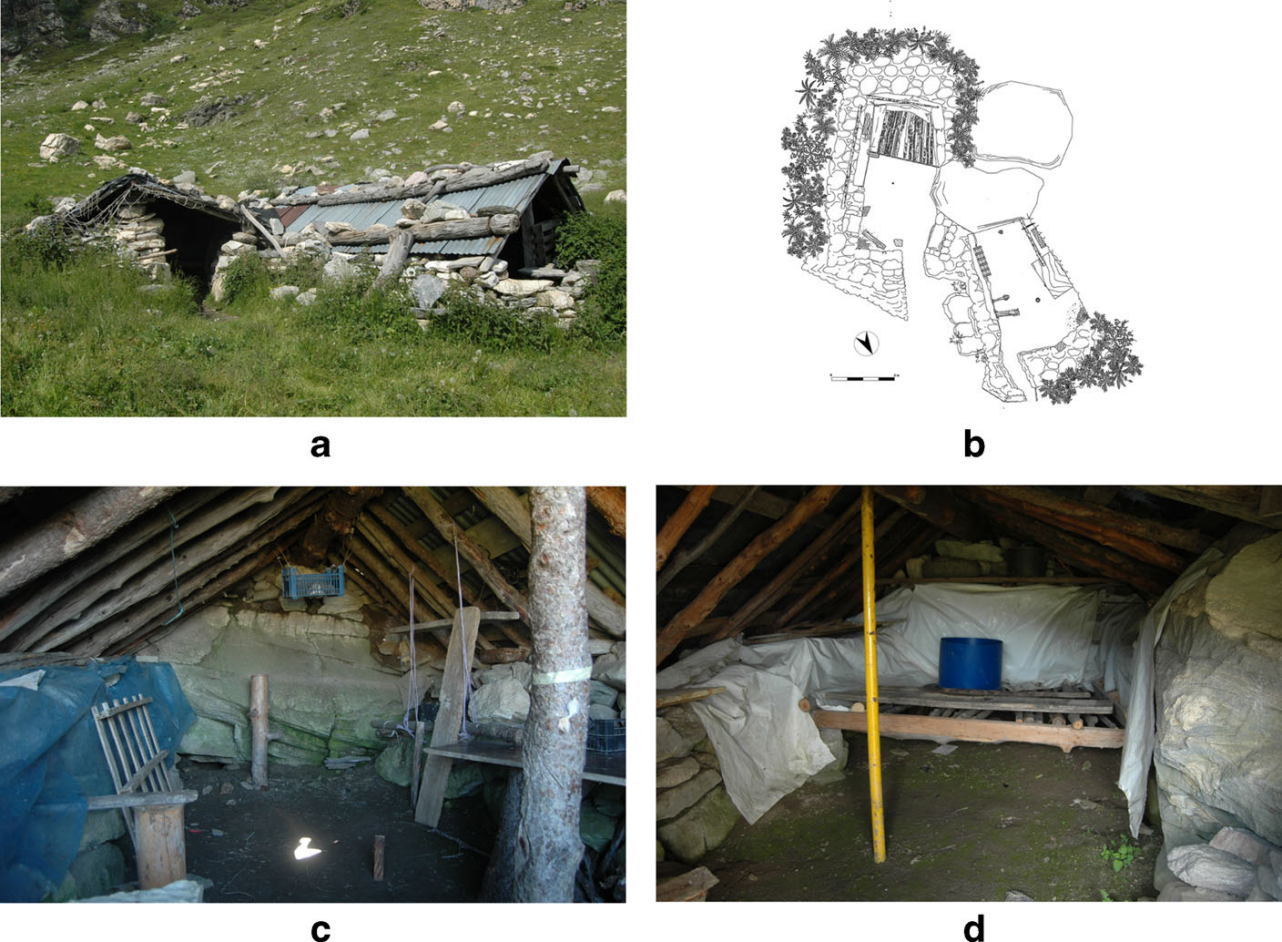
General overview of the site (**a**), plan of the site (graphics layout: Federico Panighel) (**b**), the interior of FB018A (**c**) and the interior of FB018B (**d**)

The two huts used for this research were part of the intermediate (or *secondo*) *gias* of this valley (Fig. 2a). They were located at 1937 m above sea level (asl), on a gently sloping terrace above the timber-line, close to a mountain spring and a few metres away from the cellar known as *sella Brignola*. They were recorded as FB018A and FB018B. A topographic survey of the two huts was undertaken, and the position of the artefacts and ecofacts within them was recorded. Interviews with the herder and participant observation provided important qualitative information, and enabled an understanding of the current use of these two huts, as well as of the changes that have occurred over the last few years and decades.

According to the pastoral system described above, these huts were occupied for 20–25 days every summer (in July). They were partially underground on the south side, and had low irregular dry-stone walls that were on average 0.85 m high; the overall height of the huts (including the roof) was on average 1.80 m. The two buildings were flanked on the long side and had a similar (but not identical) orientation: roughly north–south for FB018A and north-east–south-west for FB018B (Fig. 2b). The internal floors were not paved and were not level. These huts had not been built by the interviewed herder but were periodically restored by him.

The western hut (FB018A) was used as seasonal dairy (*casót del latte*) (Fig. 2c). Its internal surface was 10.7 m^2^, the entrance was on the eastern part of the north side (the short side) and the back leant against a boulder. Inside the hut, along the east and west walls, there were tables where the herder pressed, drained and shaped the curds to prepare the cheese; these tables, made of planks and pellets, rested on posts, bricks and stones. In the west internal corner, there was a fireplace, unbounded and unstructured, where the herder warmed and curdled the milk. Three posts were aligned within this building: one inside the north wall, the second in the centre of the room and the third (partially offset) at the back of the room; this last post was cut, as it had been replaced by the central one but not removed. The roof was composed of a wooden two fold structure covered by metal sheeting. On top of the roof, some objects (such as car batteries, pipes and posts) had been recycled as weights to anchor the metal sheeting against the strong winds that blow at this altitude.

The eastern hut (FB018B) was used as a dwelling (*casót del fogo*) by the herder (Fig. 2d). Its internal surface was 11.3 m^2^, and the entrance was on the northern part of the north-east side (the short side). It shared part of the boulder at the back of FB018A and part of its north-west wall was the east side of FB018A. The fireplace was in the east internal corner; as in FB018A, the hearth was unbounded and unstructured, and it was used by the herder for heating the room and cooking. The only furniture was a wooden platform, made of long poles overlain with planks, leaning on bricks and stones, which occupied a third of the internal surface (the back of the room) and was used by the herder as a bed. Most of the daily activities carried out by the herder, such as food preparation, eating and socializing, were performed around the fireplace. The roof was two folded, with a wooden structure covered by nylon fabric and metal sheeting. Nylon fabric also covered part of the internal walls to help insulate the room. Recycled objects were again used to anchor the roof to the building.

The roofs of the two huts used to be removed at the end of every summer, so that they were not damaged by the winter snow load. About 15 years previously, the herder had decided to build a permanent roof, using the same materials (posts and metal sheeting) but creating a more robust structure. This construction activity may have affected the preservation of the walls, the characteristics of the furniture and the internal surfaces.

In both huts, the fireplaces were located along the front wall (the north wall in FB018A and the north-east wall in FB018B). A hollow between the wall and the roof enabled smoke to leave the internal space.

According to the herder, the internal surface of the two huts was usually scraped clean every 3–4 years. The last scraping occurred approximately 4 years before this study. This suggested a relatively low maintenance of the huts, presumably related to their short-term use (less than a month every year). The fireplace, however, tended to be cleaned every year, at the beginning of the season.

The herder used to leave specific categories of objects in the huts when he moved to the higher *gias*, to be reused the following summer. He used to take with him only the most valuable objects or those objects that could be damaged during the winter: the cauldron, food supplies, sleeping bags, wooden rings (to shape the cheese), churn, *etc.* This was clear evidence of ‘delayed curation’, related to the seasonal abandonment and reoccupation of pastoral sites (Tomka 1993). Furthermore, most of the bulkiest waste was removed by the herder, before moving to another *gias*, and thrown into the bins provided by the municipality; therefore, there was no evidence of secondary waste in the surrounding area. Such behaviour has important consequences for the composition of archaeological assemblages at seasonal sites, as will be discussed below.

## Data Collection and Management

All the artefacts and ecofacts inside the two huts were recorded. Most of them were scattered on the internal surfaces but several had been carefully deposited within holes in the dry-stone walls or in the slots between the ridges of the walls and the folds of the roofs.

The following information was recorded for each object:

- Coordinates: local projected *x-y* coordinates in centimetres
- Size: the dimensions of length and width were recorded, but thickness was not measured because it was misleading for most of the objects recorded (*e.g.* bags, sheets and aluminium foil)
- Fragmentation: three categories of fragmentation (an indication of preservation) were used. (1) Fragment, when the original shape of the object was not clearly recognizable; (2) portion, a recognizable portion of an object, such as the handle of a mug; and (3) complete, *i.e.* a complete object. Refitting of fragments was not tested
- Material: the material that each object was made of
- Function: a description of the object and an interpretation of its function

The periodical scraping of the surface suggested that the objects recorded on the floor could be attributed to the last 4 years of occupation. The objects recorded in or on the walls, however, might pertain to a longer time span, possibly corresponding with the construction of the permanent roof (12–15 years ago).

The internal floor of the huts was kept constantly humid by the presence of the permanent roofs, which retained moisture inside the huts. Nevertheless, the surface was only moderately loose and weakly penetrable because the topsoil was quite rich in silt. Soil penetration was not tested during the survey, because only the objects visible on the surface were recorded.

The recorded spatial and attribute data were managed with GRASS 6.4 (grass.osgeo.org), an open-source geographical information system (GIS) (Neteler and Mitasova 2008). Exploratory data analyses, spatial analyses and geostatistical analyses were carried out using R 3.0 (r-project.org), a free software and environment for statistical computing and graphics (Crawley 2007) (see the “Acknowledgments” for details). Raw-data and R-code are presented in Supplementary Material.

## Objects in the Walls

Several objects were found embedded in the walls (Table 1). Most of the objects recorded were complete, and this suggested that they were stored inside the wall to be reused in subsequent years. However, the number of recorded objects was quite low: 12 for FB018A and 9 for FB018B. Most of the material culture used by the herder was removed from the huts each year, and only a small number of objects were stored. This was further confirmation of delayed curation (Tomka 1993). the archaeological implications of which will be discussed below. A small number of the recorded artefacts were not reusable objects but bulky waste, such as empty packets and lids. They may have been placed in the wall to clear the internal surfaces of the huts.

**Table 1:** Number and percentage of objects embedded in wall, divided in categories according to their fragmentation, material and function

	FB018A		FB018B	
	Number	Percentage	Number	Percentage
Total	12		9	
Fragmentation				
Fragment	0	0	0	0
Portion	3	25	1	11.1
Complete	9	75	8	89
Material				
Aluminium	1	8.3	1	11.1
Film	0	0	3	33.3
Glass	1	8.3	1	11.1
Metal	3	25	0	0
Plastic	4	33.3	3	33.3
Tin	2	16.7	1	11.1
Wood	1	8.3	0	0
Function				
Bag	0	0	3	33.3
Bottle (used as funnel)	1	8.3	0	0
Bottle of medicine	0	0	1	11.1
Bottle of soft drink	1	8.3	1	11.1
Bottle of sparkling water	0	0	1	11.1
Butter mould	1	8.3	0	0
Cap of bottle	0	0	1	11.1
Iron hook	3	25	0	0
Jar	1	8.3	0	0
Mess tin	1	8.3	0	0
Pasta packet	0	0	1	11.1
Pot	1	8.3	0	0
Tin	1	8.3	0	0
Tin cap	0	0	1	11.1
Tube	2	16.7	0	0

No predominant occurrence of specific material was evident. The function of the objects, however, provided interesting information. A butter mould stored in FB018A was a clear indication of the dairying function of this hut. A bottle transformed into a funnel (used to pour whey from the cauldron to a smaller container) and a pot (potentially used as a cauldron for smaller volume of milk) appeared to confirm this assumption. In FB018B, a pasta packet denoted the use of this hut as a dwelling. A bottle of medicine (for animals) confirmed that this hut was exploited by a herder. Interestingly, a mess tin found in FB018A could have led to a misinterpretation of the function of this hut. During the observation period, the herder was never seen having lunch or dinner inside FB018A; therefore, it could be assumed that the mess tin was used for a secondary purpose, perhaps related to milk-processing.

**Fig. 3:**
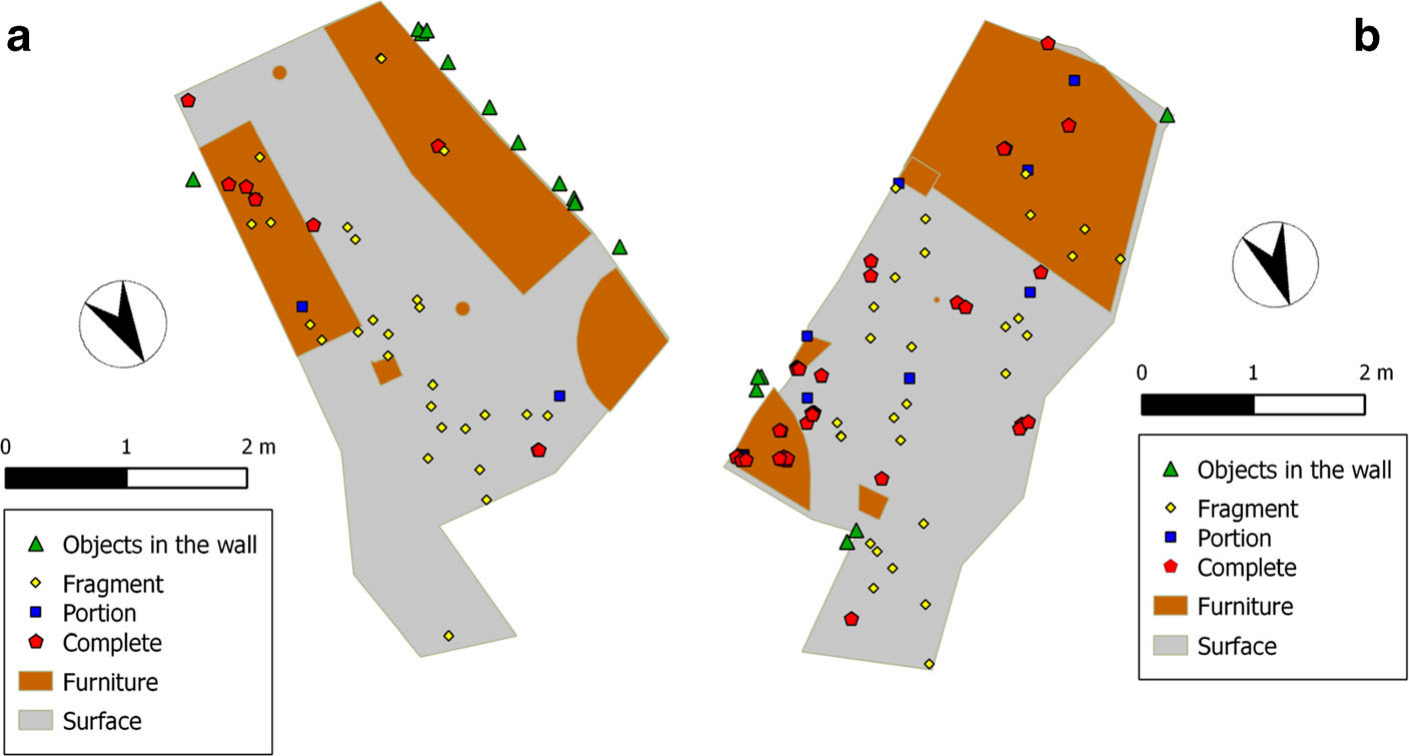
Objects in the wall and on the floor in FB018A (**a**) and FB018B (**b**)

In FB018A (Fig. 3a), 11 out of 12 objects were embedded in the west wall. There was no evident clustering, and the objects were all placed along a line from the south-west to the north-west corner. Their location seemed to be related to the position of the main table along the west wall. The herder preferred this table rather than the one placed along the east wall to shape and drain the cheese. The fireplace was positioned on this side of the building (the north-west corner) but no artefacts were found in the part of the wall close to the fireplace.

In FB018B (Fig. 3b), all the objects were strongly clustered. Three objects were clustered in the south-east wall, close to the east corner and the fireplace. Another cluster of five objects was located in the corner between the entrance corridor and the north-east wall. Three of these five objects were bulky waste, evidently stored in this area in order to clear the internal surface of the hut. An isolated object (a soft drinks bottle) was found in the west corner of the building, in the sleeping area. The presence of two clusters close to the fireplace was important as most of the herder’s daily activities were carried out around the fireplace.

## Objects on the Floor

Unlike the objects in the walls, every object on the floor could be classified as waste. The objects recorded on the floor were relatively numerous, which meant statistical methods could be applied to identify patterns. The main characteristics of the datasets are summarized in Table 2.

**Table 2:** Number and percentage of objects on the floor, divided in categories according to their fragmentation, material and function

	FB018A		FB018B	
	Number	Percentage	Number	Percentage
Total	38		74	
Fragmentation				
Fragment	27	71.1	31	41.9
Portion	3	7.9	10	13.5
Complete	8	21	33	44.6
Material				
Aluminium	1	2.6	2	2.7
Bone/teeth	0	0	2	2.7
Eggshell	0	0	1	1.4
Film	6	15.8	19	25.7
Glass	10	26.3	5	6.8
Gum	1	2.6	1	1.4
Metal	1	2.6	1	1.4
Nylon	6	15.8	13	17.6
Paper	6	15.8	15	20.3
Plastic	4	10.5	2	2.7
Pottery	1	2.6	1	1.4
Pit/seed	2	5.2	9	12.2
Function				
A5 sheet	0	0	1	1.4
Apricot pit	0	0	1	1.4
Bag	0	0	2	2.7
Bone	0	0	1	1.4
Bottle seal	1	2.6	0	0
Can	0	0	1	1.4
Candy packet	0	0	14	18.9
Cigarette butt	2	5.3	2	2.7
Coffee package	1	2.6	0	0
Cover	1	2.6	0	0
Curdle bottle	1	2.6	0	0
Eggshell	0	0	1	1.4
Foil	1	2.6	4	5.4
Glass	10	26.3	5	6.6
Gum	1	2.6	1	1.4
Iron nail	1	2.6	0	0
Label	1	2.6	0	0
Letter	0	0	1	1.4
Lighter	0	0	1	1.4
Nut	0	0	1	1.4
Nylon	6	15.8	11	14.9
Package	4	10.5	6	8.1
Paper	1	2.6	5	6.6
Pasta packet	0	0	2	2.7
Peach pit	1	2.6	6	8.1
Pine nut	0	0	1	1.4
Plastic	1	2.6	1	1.4
Porcelain	1	2.6	1	1.4
Salame label	1	2.6	0	0
Seed	1	2.6	0	0
Sheep tooth	0	0	1	1.4
Snack packet	2	5.3	2	2.7
Wire	0	0	2	2.7

Thirty-eight objects (artefacts and ecofacts) were recorded in FB018A (3.5 objects per m^2^) and 74 in FB018B (6.5 objects per m^2^). The number (and the density) of objects recorded in the dwelling was double that of the dairy. This significant difference could be attributed to the different functions and the different intensity of exploitation of the two huts. There was no doubt that the dwelling FB018B, where most of the herder’s daily activities took place, was more intensively exploited than the dairy FB018A, where only a specialized production activity was carried out. This seemed to have had an impact on the number of objects scattered on the surface; in other words, there appeared to be a direct correlation between the intensity of exploitation and the number of objects recorded within the huts. The analysis of materials highlighted the fact that some ecofacts were strictly related to food consumption, such as bones, teeth and eggshells, and occurred exclusively in FB018B. This difference was clearly explained by the different function of the two huts: in FB018B the herder prepared and ate his meals, while FB018A was almost exclusively exploited for milk-processing. Similar and quite high values of nylon (15.8 % for FB018A and 17.6 % for FB018B) in the two huts were the result of the progressive degradation of the nylon insulation of the roofs.

The different function of the two huts was also suggested by the artefacts recorded in FB018B: cans (absent in FB018A), sweet packets (18.9 % in FB018B and 7.5 % in FB018A) and pasta packets (absent in FB018A). Various food-associated artefacts did occur in FB018A but the sum of all the food-associated artefacts and ecofacts showed that 41.9 % of the entire FB018B dataset was related to food processing and consumption, while for FB018A only 13.2 % of the objects were associated with food; the chi-square test suggested that the difference between these proportions of food-associated objects was statistically significant (*χ* ^2^ = 9.5069, df = 1, *p* = 0.0020). However, a bias in the dataset cannot be excluded: the number of complete objects was higher in FB018B than in FB018A, and this could increase the recognisability of the function of objects for the dwelling, thus decreasing the reliability of the comparative analysis. Nevertheless, the objects placed in the wall led to the same functional interpretation (see above) and the effect of dimensional inconsistency between the two huts can be underestimated.

A bottle of rennet was the only object found on the floor of FB018A that referred to the specific function of this hut. Its presence was consistent with the objects found in the wall.

## Point Pattern Analysis

The aggregation and segregation of the objects on the internal surface of the huts were analyzed. These spatial phenomena can be seen as the result of processes of discard and fragmentation that mirror specific intra-site activities. Preliminary observations of the placement of artefacts and ecofacts suggested that different levels of clustering may have occurred at different scales. Therefore, an *L*-function, Besag’s transformation of Ripley’s *K*-function (Besag 1977; Ripley 1976). was used, because it enables the identification of clustering at different distances. Both *K* and *L*-functions are widely applied in archaeology, to investigate spatial patterns at intra-site and landscape scale (Bevan and Conolly 2006; Eve and Crema 2014; Orton 2004; Palmisano 2013; Vanzetti *et al.* 2010).

They are usually visualized on a plot, with the expectation under randomness ($L(r)=r$) drawn as a line. A value below the line indicates a regular pattern; a value above the line indicates a clustered pattern. $L(r)$ values were estimated for 9999 random Monte Carlo-simulated patterns and compared with the values estimated for the dataset (Baddeley *et al.* 2014). In order to reduce the bias of the edge effect, a Ripley’s isotropic correction was implemented (Ohser 1983).

**Fig. 4:**
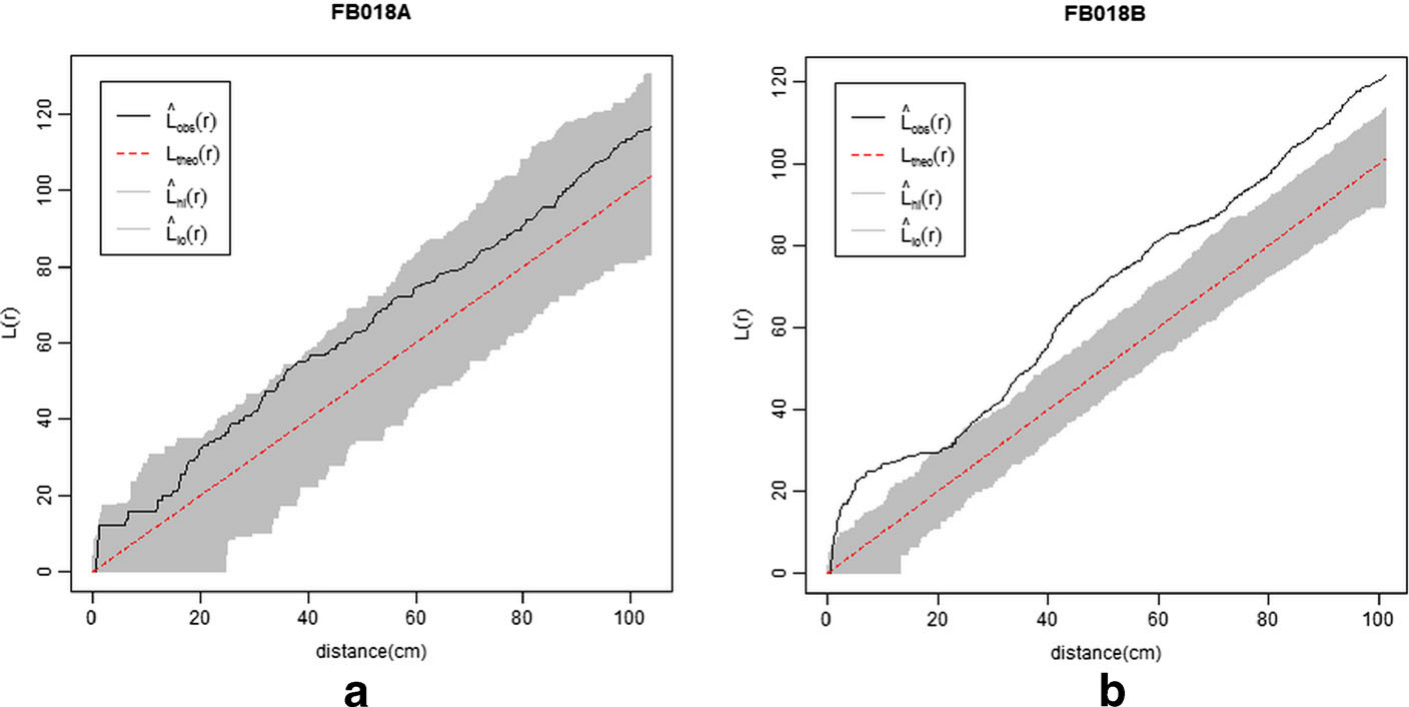
Plots of the *L*-functions for FB018A (**a**) and FB018B (**b**) datasets. The envelope has been generated from Monte Carlo simulation (9999 iterations) under CSR

No significant degree of interaction could be assumed for the objects recorded within FB018A (Fig. 4a). According to this result, the null hypothesis of complete spatial randomness of objects in FB018A cannot be rejected.

A visual assessment of the object pattern in FB018A (Fig. 3a) showed that most of the fragments were scattered along a line between the north wall and the final part of the east wall, while portions and complete objects were placed along the walls. This might suggest that their spatial organization had been affected by post-depositional processes. Noteworthy also is the absence of objects within or around the fireplace. As a matter of fact, the fireplace of FB018A was only used to warm up the milk and it was an activity area of secondary importance.

**Fig. 5:**
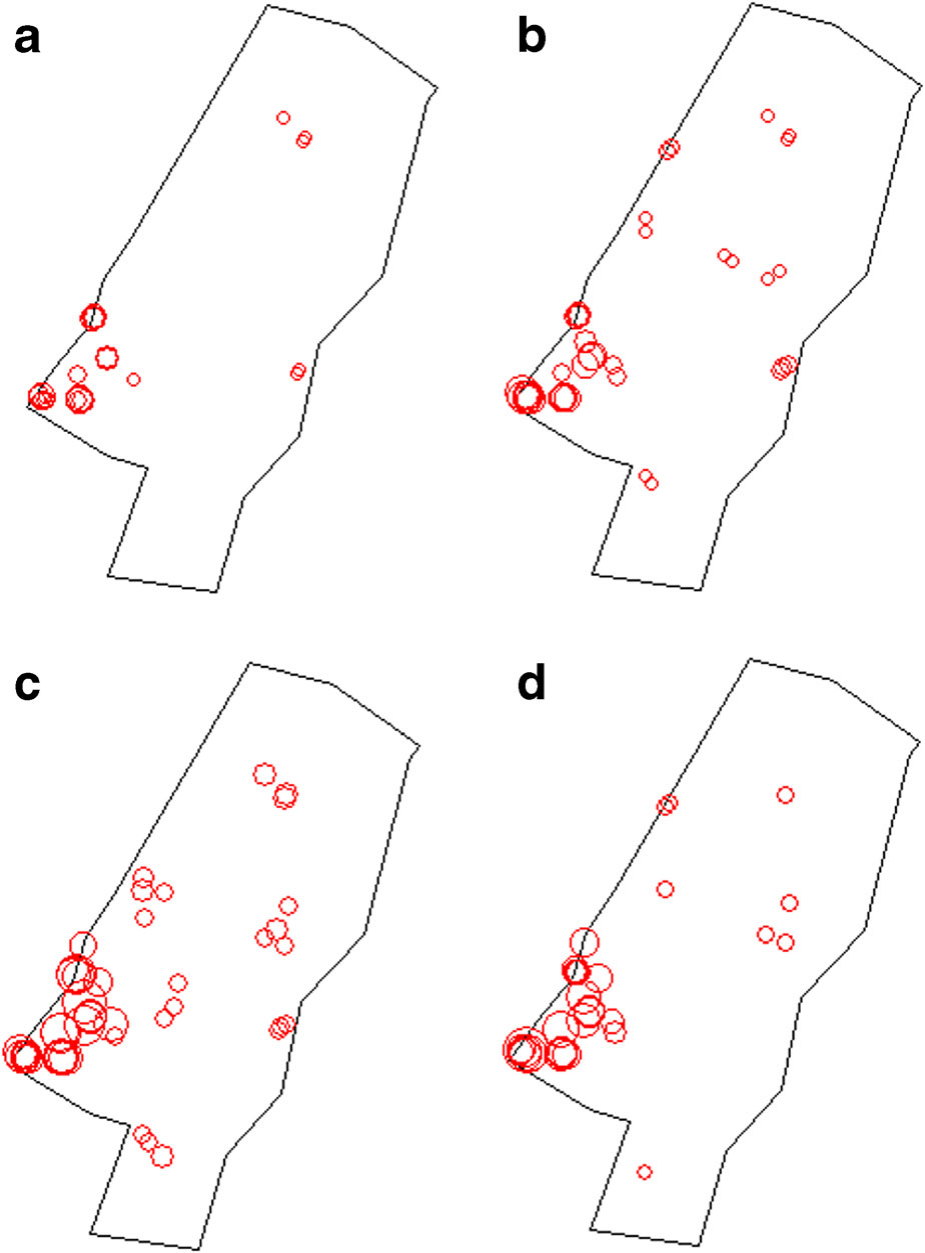
Plots of the local *L*-function values (above the expectation of CSR) for FB018B dataset at different bandwidths: **a** 5 cm, **b** 15 cm, **c** 30 cm, and **d** 40 cm. Each *circle* corresponds to an object recorded on the floor, and its diameter is proportional to the *L*-function value estimated for the object

In FB018B, instead, artefacts and ecofacts were significantly clustered at almost every distance (Fig. 4b); namely, every $L(r)$ value estimated for our dataset was higher than the highest $L(r)$ value of the envelope for (almost) every distance. Since the envelope is created under complete spatial randomness (CSR), the spatial process underlying the point pattern is assumed to be homogeneous and stationary for the entire area. Local *L*-function was estimated for every object at different bandwidths (5, 15, 30 and 40 cm), and the plots (Fig. 5) showed that the highest *L* values aggregate within and around the fireplace. This evidence is not surprising: as already mentioned, the fireplace was the core of FB018B. Around the fireplace, most of the daily activities of the herder were undertaken; therefore, it was more likely that the objects were discarded here than anywhere else in the hut. This suggested that the spatial process underlying the point pattern (*i.e.* object discarding) was not homogeneous, as there was a first-order effect (*i.e.* proximity to the fireplace) that influenced the variation of the intensity of points (*i.e.* density of objects) at different locations within the study region (Bevan *et al.* 2013).

**Fig. 6:**
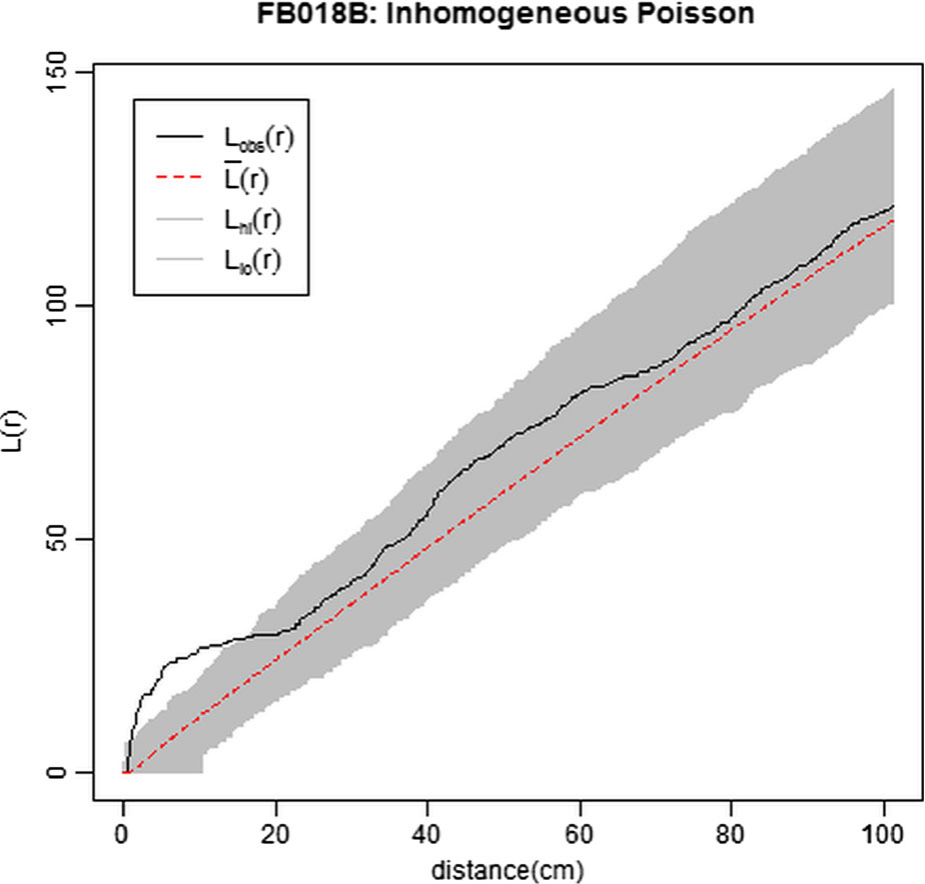
Plots of the *L*-function for FB018B dataset. The envelope has been generated from Monte Carlo simulation (9999 iterations) of the point process model

In order to confirm the influence of the fireplace on the object pattern and to assess whether there were other first- or second-order effects operating, an inhomogeneous Poisson point model fitting Euclidean distance from the east corner of the structure (the fireplace location) was created. In this model, the proximity to the fireplace was assumed to account for the density of objects, namely the density of objects was expected to decrease when the distance from the fireplace increased. Akaike Information Criterion was used for comparing the fitted model with a homogeneous point process model. The smaller AIC and the higher Weight favoured the fitted (AIC = 1203.3, *w =* 1) over the homogeneous (AIC = 1234.5, *w* < 0.001) model, thus confirming that the intensity of the point process varied as a function of the distance from the fireplace. *L*-function was applied to our dataset and to 9999 Monte Carlo-simulated patterns conditioned on the spatially inhomogeneous model. The plot (Fig. 6) showed significant clustering of the objects at short distances (5 to 15 cm). This result suggested that proximity to the fireplace did not explain completely the identified spatial aggregation, and that other first- or second-order effects influenced the intensity of the point process.

Plots of local *L*-function values presented before (Fig. 5a, b) showed aggregation of object scattered on the floor for short distances (5 and 15 cm). Visual assessment of the spatial organization of fragmentation categories (Fig. 3b) suggested that the aggregation of fragmented objects can be attributed to *in situ* breakage and that complete objects are clustered along the walls, near the central post and underneath the platform. These spatial patterns might account for the residual aggregation suggested above, and seemed to indicate the existence of post-depositional disturbances that caused fragmentation of objects or secondary displacement from their primary location.

## Trampling and Spatial Autocorrelation

The assumption about fragmentation and secondary displacement of objects led to the analysis of one of the main processes that might have affected the patterns of objects within FB018A and FB018B: trampling. The spatial distribution of fragmentation categories (Fig. 3) suggested two processes: (1) actual trampling, causing the breakage of objects in the most intensively used areas of the building; (2) displacement (through deliberate tossing or kicking out of the way) of bulkier objects to more peripheral areas. The abundance of complete objects clustered around and within the fireplace in FB018B suggested that this area was not (or only weakly) affected by trampling.

As mentioned above, the effects of trampling on archaeological assemblages have been widely investigated through experimental archaeology. The main aims of these experiments were to identify the degree of vertical migration of objects across stratigraphic units, recognize the damage caused by trampling on artefacts and ecofacts and discriminate these post-depositional damages from other evidence (*e.g.* butchery cut marks on animal bones). Some of these experiments have provided interesting inferences about the horizontal displacement of objects (Gifford-Gonzalez *et al*. 1985, pp. 808–810; Villa and Courtin 1983, pp. 277–278), although only Nielsen (1991) has proposed a detailed analysis of this phenomenon. From the experimental data collected, Nielsen surmised the existence of three different patterns of horizontal migration, correlated with the size of the objects: very small objects are soon embedded in the soil, thus reducing their movement; small and medium objects are randomly displaced by trampling on the surface, and their original spatial pattern is rapidly obscured; if trampling continues, small and medium objects accumulate in marginal zones (along the walls in enclosed spaces) or around large objects; bulky objects are soon moved (kicked) aside, into marginal zone (Nielsen 1991, p. 492). The result should be ‘a “marginal zone” characterized by a high proportion of bulky artifacts, and a “traffic zone” with small- and medium-size items randomly scattered and very small ones buried close to their original spot of deposition’ (Nielsen 1991, p. 500). Regarding the size distribution of assemblages (Nielsen was particularly focused on ceramic assemblages), he suggested that increasing trampling would result in an increasing positive skewness of the distribution that would progressively approximate a Poisson distribution (Nielsen 1991, pp. 493–495).

**Fig. 7:**
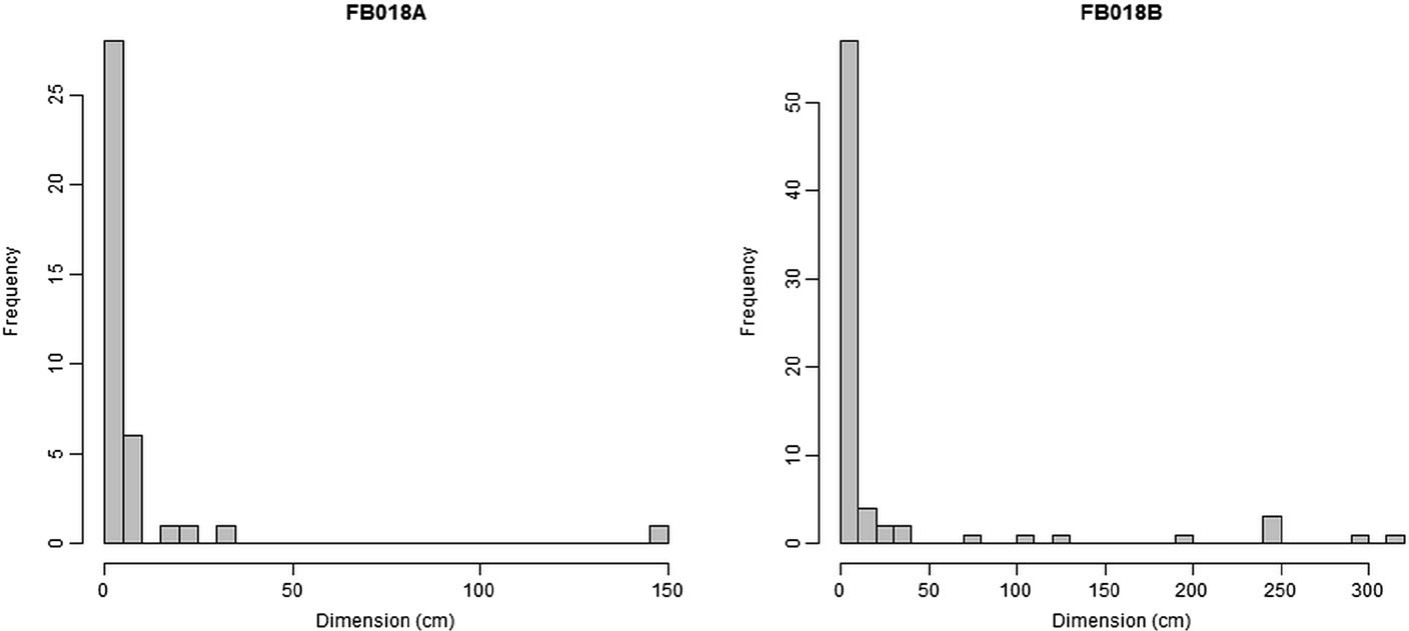
Histogram of object sizes in FB018A and FB018B

Nielsen’s (1991) inferences seem to be confirmed by the patterns identified in FB018A and FB018B. First of all, strongly and positively skewed histograms of the size distribution of objects indicated an intense and extended trampling on the internal surfaces of the two huts (Fig. 7). However, this skewed distribution might be exaggerated by the voluntary removal of large objects (delayed curation). The spatial organization of fragmentation categories on the surfaces, as pointed out before, seemed to follow the pattern proposed by Nielsen (1991). complete objects along the walls or in marginal areas and fragmented objects scattered on the surface. However, more specific investigations of the actual size values were necessary to confirm this visual assessment.

According to Nielsen’s (1991) inference, objects of similar size are expected to be closer to each other than objects of different sizes; furthermore, a difference in size is supposed to increase with distance. These assumptions reflect the first Tobler’s law of geography (Tobler 1970). stating that the correlation between spatial values tends to decrease when distance increases. This property of spatial features is known as spatial autocorrelation, and is the basic concept of geostatistics. It is worth pointing out that other spatial processes might be accounted for intra-site autocorrelation of object size, for instance spatially segregated activities producing primary refuse of different sizes. However, the statistical analysis of spatial interactions between points and the visual assessment of the spatial pattern of fragmentation categories, presented above, led to exclude this possibility.

In this study, Moran’s *I* correlogram and empirical variogram were used to estimate autocorrelation in FB018A and FB018B datasets.

The use of Moran’s *I* statistic is quite common in archaeological research (Kvamme 1990; Premo 2004). The coefficient (*I*) estimation is based on the spatially weighted cross-products of deviations from the mean calculated on a specified variable at all pairs of points in turn; *I* values range from −1 (perfect dispersion) to +1 (perfect correlation), and a zero value indicates a random pattern (Cliff and Ord 1973; Moran 1950). The difference of observed *I* values from the expected values can be statistically tested using assumption of normality. In a Moran correlogram, Moran’s *I* values are plotted as a function of distances among objects, in order to discover changes in spatial dependency at different scales (Legendre and Legendre 2012, p. 715–721).

In a variogram, a value $\widehat{\gamma}(h)$ is estimated by calculating the sum of the squared difference of paired points separated by a lag, obtaining half the average for all the observations divided by the same lag (Chilès and Delfiner 1999, pp. 29–150; Lloyd and Atkinson 2004, p. 153). If the analyzed values are spatially autocorrelated, the semi-variance (*γ*) increases with separation distance (*h*). Bounded models reach a sill (finite variance) after which semi-variance and distance are independent. A variogram can be calculated for all directions (omnidirectional) or for a specific direction (directional), thus enabling the identification of anisotropy or spatial variation. A variogram map, a matrix of *γ-*values for *x* and *y* separation distances (*h*), is also a useful and intuitive tool for assessing spatial anisotropy. Variogram has been used in archaeology for spatial interpolation and predictive modelling (Lloyd and Atkinson 2004; Rondelli *et al*. 2014; Wells 2010). and has been applied recently to address spatial anisotropy in archaeological assemblages (Bevan and Conolly 2009; Markofsky and Bevan 2012).

**Fig. 8:**
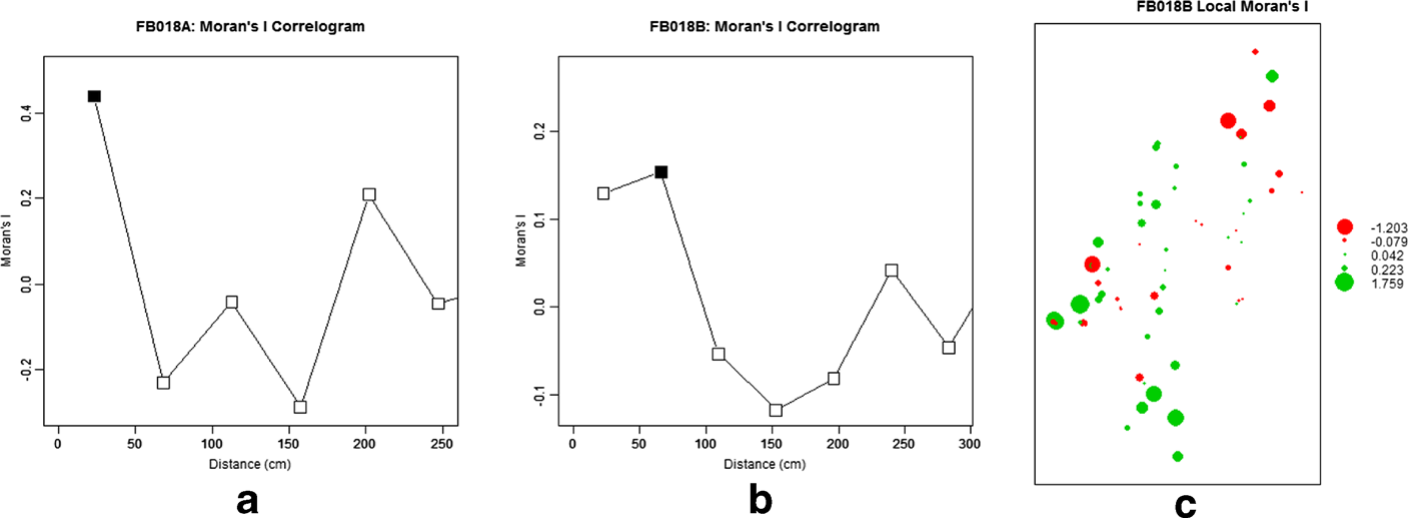
Plots of the Moran’s *I* correlograms for FB018A (**a**) and FB018B (**b**) datasets (the *black square* indicates statistically significant *I* values, *p < 0.05*) and plot of the local Moran’s *I* values for FB018B dataset (**c**)

Moran’s *I* was estimate for the size of objects in the two huts. As the size values were heavily right-skewed (see Fig. 7), they were transformed using a natural logarithm function. The number of distance classes has been computed using the Sturges method. Coefficients for the larger distance values (>250 cm in FB018A, >300 cm in FB018B) were not considered. Spatial correlogram of Moran’s *I* values for FB018A (Fig. 8a) showed significant positive autocorrelation for the shortest distance (23 cm). For FB018B (Fig. 8b), instead, correlogram showed no significant autocorrelation for the first distance class (22 cm) but significant positive autocorrelation for the second (65 cm).

The lag of empirical variogram was set at 13 cm, and the threshold distance was calibrated based on half of the approximate maximum width of the huts: 135 cm for FB018A and 120 cm for FB018B. The attribute was the size of the scattered objects. As for the Moran’s *I* statistic, the size values were transformed using a natural logarithm function.

**Fig. 9:**
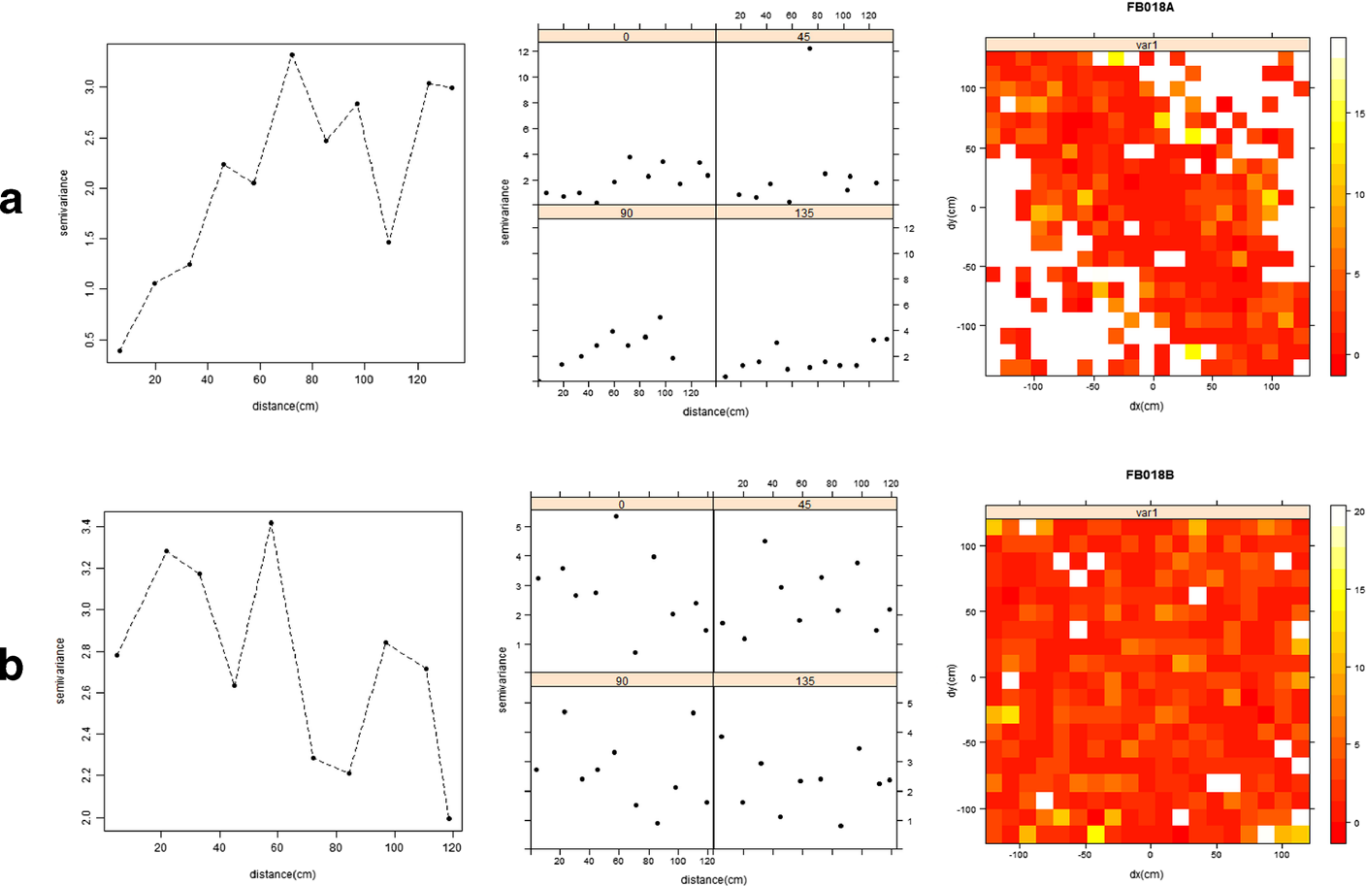
Omnidirectional variogram, directional variogram and variogram map of (natural logarithm of) object size in FB018A (**a**) and FB018B (**b**)

For FB018A, the omnidirectional empirical variogram (Fig. 9a), although jagged (for the limited number of data considered), suggested a positive autocorrelation; after 50 cm semi-variance and distance seemed to be independent. This result was quite consistent with that provided by Moran’s *I* correlogram. Some of the values of the 135° directional variogram were equal or lower than the omnidirectional values, and this suggested a slight spatial anisotropy for this direction. However, the variogram map was not clear, and anisotropy could not be confirmed. For FB018B, the omnidirectional variogram did not show any spatial autocorrelation, and this contrasted with the results of Moran’s *I* statistic. Directional variograms and a variogram map did not provide evidence of anisotropy (Fig. 9b).

The objects scattered inside FB018A proved to be significantly autocorrelated within short distances. This confirmed Nielsen’s (1991) experimental inference, that trampling produces a secondary displacement of objects according to their size. No clear evidence of spatial anisotropy was provided for FB018A. This might confirm Nielsen’s assumption that trampling was omnidirectional or random.

Moran’s *I* correlogram and variogram returned misleading and contrasting results for FB018B. Omnidirectional variogram did not provide any evidence spatial autocorrelation. Significant positive autocorrelation was instead highlighted by Moran’s *I* correlogram, although not for the shortest distance. The plot of local Moran’s coefficient values (Fig. 8c) showed a concentration of high and low values in two sectors of the hut corresponding to the fireplace and the platform. This suggested that depositional and post-depositional processes occurring in these intra-site areas influenced the spatial relationships between object sizes, thus preventing the identification of possible trampling effects.

It could be argued that the difference between the two assemblages resulted from the different strategies and intensities of exploitation of the two huts. FB018A (the dairy) experienced a less intensive and more specialized exploitation than FB018B, which was used as a dwelling and main living area by the herder. Different activities took place within FB018B and had different consequences on the size, fragmentation and spatial location of the objects recorded. Trampling was the main factor that influenced the displacement of objects in FB018A, while its effects on the FB018B dataset were nuanced and hidden by other spatial processes.

## Discussion: Implications for Archaeology

Based on the current data and previous inferences, it appears that the deposition of objects inside the two huts reflects the complex processes that have occurred (and were still occurring) during the last few years. The interplay between information provided by ethnographic interviews and the results of spatial analysis can be used to provide archaeological insights that further our understanding of seasonal sites and intra-site assemblages.

## Site Function

One of the main interpretative problems for seasonal archaeological sites, and especially for pastoral sites, is identifying their function. It is not clear which markers can provide significant indicators of how a structure was used and for what purpose (see Chang and Koster 1986). The ethnoarchaeological case study presented here provided some valuable hints to tackle this issue, and it might constitute the basis for future investigations.

The two huts were of approximately the same shape and dimensions, and had been built using the same technique. Therefore, their structural characteristics did not discriminate their function. The internal furniture, wooden tables (to process the cheese) in FB018A and a wooden platform (used as a bed) in FB018B, provided evidence of the different uses of the internal space. Post-holes, stones and bricks would be the only archaeological signs of the existence of the tables and platform. The presence of complete objects scattered or clustered along the walls could suggest indirectly the presence of furniture inside the huts, but it would be very difficult to make any confident inference from this scant evidence.

The ratios of functional and material categories of objects scattered inside the buildings (and in the walls) did provide important indications of the use of the huts. Objects related to food processing and food consumption were abundant in FB018B and scarce in FB018A, which confirmed that the first hut was used as a living area while the second had another and more specialized function. Some of the objects found in FB018A (a rennet bottle, funnel and butter mould) also suggested the use of this hut as a dairy. The overall number of objects recorded in FB018B (74) and in FB018A (38) seemed to confirm a more intensive exploitation of the former and the different uses of the two huts. The clustering of objects within and around the fireplace in FB018B and the absence of objects around the fireplace in FB018A provided further evidence of differences between the two huts.

It is worth noting, however, that delayed curation, namely the removal of valuable and bulky objects from the site (Tomka 1993). might affect the reliability of the archaeological record for the interpretation of site function. Even though some objects enabled a basic functional discrimination, most of the objects that would have been clearly related to site exploitation had been removed by the herder. This also meant that some of the activities carried out within the huts were under- or not represented, because they were less archaeologically ‘visible’ and because they were affected more by delayed curation: for instance, heating the milk in FB018A (the cauldron and the structure it hung from had been removed) and sleeping in FB018B (the sleeping bags and mattresses had been removed). The permanent roof might also have had a role in the preservation of the assemblages and their patterning: it is worth considering whether similar preservation can be expected for a structure with a removable roof.

Another interesting aspect that emerged from this analysis was the complex relationship between the frequency of exploitation and the number of objects at a seasonal site. This issue has been addressed by several ethnoarchaeological projects (see, for instance, O’Connell 1987, pp. 74, 105), and a direct correlation between these factors has been questioned. The two pastoral huts used for the current research provided interesting data relevant to this topic. The abundance of objects helped discriminate between the functions of the two huts. Therefore, it can be assumed that the intensity of exploitation, rather than the frequency of exploitation (or the duration of occupation), influences the assemblage size at a seasonal site, as FB018A and FB018B were used during the same period (20–25 days in July) but had different functions. This is only a preliminary suggestion, but it could provide a new angle on the issue of visibility and recognizability of seasonal sites (Cribb 1991, pp. 65–83; Gifford and Behrensmeyer 1977).

## Intra-site Activity Areas

As pointed out in the introduction, the main aim of ethnoarchaeological research on intra-site patterns has been the identification of activity areas. The current ethnoarchaeological research was driven by this aim as well. Although the two huts themselves could be recognized as two areas of activity within the same site (a residential area and a productive area), secondary activity areas could also be identified within each hut. In this case study, the two huts are better described as functional units (see Hillier and Hanson 1984) with embedded activity areas.

The location of objects inside sites is supposed to reveal (directly or indirectly) the location of activity areas. First of all, it is worth highlighting the low level of maintenance documented in the two huts analyzed. This low maintenance, in combination with the short period of exploitation, had a decisive role in the preservation of spatial associations between objects. Therefore, reliable identification of activity areas seems to be inversely proportional to the degree of maintenance of the surface. Other ethnoarchaeological projects focused on mobile groups have arrived at similar conclusions (Binford 1978, p. 357; O’Connell 1987, pp. 91–92; Simms 1988, p. 208). Several objects were found embedded in the walls of the huts. Their location was affected neither by post-depositional displacement (trampling, see below) nor by maintenance, but was the direct consequence of the deliberate positioning of items for different purposes (mostly re-use but also surface clearing) (Binford 1978, p. 346). Activity areas can be inferred from their spatial patterns but they might be biased by delayed curation (see above). Moreover, in an archaeological context, they are expected to be repositioned by the collapse of the walls. Preservation and deposition of the objects on the floor and in the walls might also be influenced by the presence of a permanent roof. It is debatable whether and how the assemblage might vary with a removable roof.

In FB018B, most of the objects in the wall were placed around the fireplace. Furthermore, most of the objects on the floor were clustered within the fireplace or the surrounding surface (a semicircle with a 60-cm radius). The consistency of the two datasets and the ethnographic observations confirm that the fireplace was the main activity area of this hut. The clustering of objects within and around the fireplace was caused by dropping and tossing, suggesting that this part of the assemblage was primary refuse (Binford 1978, pp. 345–348). Ethnographic observations revealed that the platform at the back of the hut was another important activity area: the sleeping area. Interestingly, however, only one object was embedded in the wall, and the position of the few objects under the platform seemed to be mainly the result of secondary displacement (trampling, see below). The central sector of the hut was a transition area between the primary activity area (living) and the secondary activity area (sleeping). Therefore, it experienced most of the trampling, as discussed further below.

A similar spatial organization can be suggested for FB018A. Ethnographic observations and the positioning of objects in the walls suggested that the tables leaning on the east and west walls were the main activity areas of the hut (especially the west wall). The scattering of objects on the internal floor, however, seemed to have been probably caused by trampling (see below), and the central area of the hut could be interpreted as a transition area. The fireplace corresponded to another activity area, even though no object was documented within or around it. According to these results, the two huts could be divided into three different areas.

- The primary activity area(s), where most of the activities were carried out and most of the objects were placed (on the floor and in the walls in FB018B, in the walls in FB018A)
- The secondary activity area, where specific activities took place but few material traces of these activities were identified
- The transition area, the area that connected the main activity area to the secondary activity area, and where possible traces of trampling were identified

The proposed division highlights an unexpected regularity in the use of intra-site space, despite the difference in function between the two huts. Several activities were carried out in the primary activity areas (food processing, eating and relaxing in FB018B; milk processing, cheese shaping and cheese drying in FB018A), while the secondary activity areas seemed to be more ‘specialized’ and less exploited (sleeping in FB018B and heating milk in FB018A).

Other ethnoarchaeological projects that have focused on seasonal sites have pointed out the difficulty in identifying actual activity areas (O’Connell 1987, p. 105; Simms 1988, pp. 207–208; Yellen 1977); this may be attributed to the medium/long-term exploitation of the sites (directly proportional to the degree of surface maintenance) and/or the low archaeological visibility. Binford, instead, revealed a certain degree of spatial segregation at the Mask site, recording four different activity areas and assuming that: ‘the degree that activities will be spatially separated at any one time can be expected to vary with the number of different activities simultaneously performed by different persons’ (Binford 1978, p. 354). He suggested that some activities interfere with others and therefore cannot be carried out in the same place and at the same time. Comparable conclusions have been provided by Cribb (1991, p. 129, 176) for nomadic pastoral sites. The higher aggregation of activities evidenced in FB018A and FB018B can therefore be attributed mainly to the presence of a single herder. As he worked alone, he could not perform different activities simultaneously, he only needed to segregate the tasks that could not be performed in the same place (*e.g.* heating milk and shaping cheese in FB018A, and food processing/consuming and sleeping in FB018B) or that required additional space (the tables in FB018A). This is an indirect confirmation of Binford’s (1978) suggestion.

## Intra-Site Mobility

Ethnoarchaeological research on intra-site patterns has often perceived trampling as a post-depositional disturbance of the archaeological assemblage, and has never addressed this issue properly. Experimental archaeology has analyzed the effects of trampling with quantitative methods and has provided interesting results. However, these experiments have evaluated the consequences of short-term high-intensity trampling, whereas seasonal archaeological sites usually experienced medium/long-term low-intensity trampling. Therefore, quantitative analysis of trampling within FB018A and FB018B was considered important to provide reliable analogical models for archaeological interpretation.

Trampling seemed to be the main (or only) process that determined the dispersion of artefacts and ecofacts on the internal surface of FB018A. The primary activity area (see above) of this hut could be inferred from the objects embedded in the wall rather than from those scattered on the floor near and under the tables. The position of these objects, instead, was the result of secondary displacement (throwing or kicking aside) of bulky waste that was then trapped under the tables and protected from fragmentation. Although trampling directionality could not be identified, the linear patterns of fragmented objects in FB018A (Fig. 3a) were consistent with the main axis of the building (north–south). This suggested that the intensity of trampling can be spatially isotropic within the structure, but is partially constrained by the location of the entrance, the position of the central post and the location of one or more specific targets.

Spatial interaction between objects suggested that two different spatial phenomena co-existed in FB018B: fragmentation and displacement of objects in the transition area and clustering in the main activity area (the fireplace). Autocorrelation analysis, though, did not provide any clear evidence of trampling. The effects of this post-depositional process were evidently hidden by the effects of other complex spatial processes, related to the intensive use of the hut. Objects aggregated within and around the fireplace were only weakly affected by trampling, thus keeping their original size and position.

The current data provide interesting insights for archaeology. The effects of trampling in the intra-site archaeological record seem quite predictable. In sites with more intense exploitation, several spatial processes take place and the activity areas are outside the traffic zone, therefore less affected by trampling. In contrast, archaeological assemblages in more specialized sites with low-intensity exploitation are completely displaced by trampling; very few objects are discarded in the activity areas, and are soon trampled or voluntarily and involuntarily moved aside.

## Conclusions

The application of quantitative methods (spatial analysis and geostatistics) to the study of two pastoral huts in the Italian western Alps (Table 3), as well as the interplay between these methods and ethnographic information collected in the field, has provided interesting insights which can enhance the interpretation of archaeological seasonal sites. Some of the inferences can also be applied to permanent sites. Specific archaeological markers (aggregation/segregation, object types, *etc.*) enabled the discrimination of a residential site and a site with a different and more specific function. The dimension of the assemblage depends on the intensity of exploitation rather than on the duration of occupation, while the assemblage composition depends on delayed curation. Activity areas are recognizable in sites with a high intensity of exploitation, and the spatial aggregation of activity areas is inversely proportional to the number of people exploiting the site (even though the spatial incompatibility of different tasks and the dimension of the site represent further constraints). A traffic zone or transition area, highly affected by trampling, connects two or more activity areas, and its directionality is partially constrained by the location of these activity areas, the presence of barriers and the location of the access point to the site. This spatial organization of activity areas and traffic zone resembles concepts used in access analysis (Foster 1989; Hillier and Hanson 1984; Mackie 2014) to analyze the social delineation and use of intra-site space. Access analysis has usually been applied to complex structures, where different rooms show different levels of permeability and interconnections. The two huts analyzed in this study were simple but the analysis of object clustering and scattering enabled the inference of spatial segregation or integration (*i.e.* symmetry/asymmetry) and accessibility (*i.e.* distributedness/nondistributedness) between different zones within them (Hillier and Hanson 1984, p.148). This spatial organization was related to the different function of the two huts.

**Table 3:** Summary of the main outputs of the spatial and geostatistical analysis on the assemblages of the two huts

Analysis	FB018A	FB018B
*L*-function	Homogeneous: no significant spatial interaction at any distance	Inhomogeneous: significant spatial interaction at short distances (5–15 cm)
Moran’s *I* correlogram	Significant positive autocorrelation for the first distance class	Significant positive autocorrelation for the second distance class
Omnidirectional variogram	Evidence of positive spatial autocorrelation	No evidence of spatial autocorrelation
Directional variogram	No evidence of spatial anisotropy	No evidence of spatial anisotropy

It is worth pointing out that the periodical scraping of the internal surfaces resulted in the loss of most of the intra-site artefacts and ecofacts, thus limiting our inferences to the last few years of use of the huts. This suggests that, in seasonal archaeological contexts, most of the interpretations based on intra-site patterns might refer exclusively to the last occupation phase before the definitive abandonment of the site.

This study showed that spatial point pattern and autocorrelation analysis enable more reliable and generalizable inferences to be made about the behavioural causes of depositional and post-depositional processes in archaeological sites. Other ethnoarchaeological projects have recently experimented with similar quantitative methods in intra-site contexts (see Rondelli *et al*. 2014). suggesting that the interaction between these methods and ethnographical observations can provide crucial interpretative tools for our understanding of archaeological contexts. Therefore, a wider and more sophisticated use of spatial analysis and geostatistics will enable ethnoarchaeology to regain the important role it had for the interpretation of intra-site archaeological spatial patterns.

## Electronic supplementary material

Below is the link to the electronic supplementary material.

ESM 1 (TXT 1 kb)

ESM 2 (TXT 3 kb)

ESM 3 (TXT 2 kb)

ESM 4 (TXT 4 kb)

## References

[CR1] Baddeley A, Turner R (2005). Spatstat: an R package for analyzing spatial point patterns. Journal of Statistical Software.

[CR2] Baddeley A, Diggle PJ, Hardegen A, Lawrence T, Milne RK, Nair G (2014). On tests of spatial pattern based on simulation envelopes. Ecological Monographs.

[CR3] Bartoń, K. (2015). Package ‘MuMIn’: multi-model inference. R package version 1.15.1. https://cran.r-project.org/web/packages/MuMIn/index.html.

[CR4] Besag J (1977). Discussion of Dr Ripley’s paper. Journal of the Royal Statistical Society, Series B.

[CR5] Bevan A, Conolly J, Lock G, Molyneaux B (2006). Multiscalar approaches to settlement pattern analysis. Confronting scale in archaeology: issues of theory and practice.

[CR6] Bevan A, Conolly J (2009). Modelling spatial heterogeneity and nonstationarity in artefact-rich landscapes. Journal of Archaeological Science.

[CR7] Bevan A, Crema E, Li X, Palmisano A, Bevan A, Lake M (2013). Intensities, interactions and uncertainties: some new approaches to archaeological distributions. Computational approaches to archaeological spaces.

[CR8] Binford L (1978). Dimensional analysis of behavior and site structure: learning from an Eskimo hunting stand. American Antiquity.

[CR9] Bivand R, Piras G (2015). Comparing implementations of estimation methods for spatial econometrics. Journal of Statistical Software.

[CR10] Carr C (1984). The nature of organization of intrasite archaeological records and spatial analytic approaches to their investigation. Advances in Archaeological Method and Theory.

[CR11] Casanova O, Salsa A (2002). Ambiente naturale e pastorizia nelle Alpi Marittime. Pastorizia, transumanza e segni dell’uomo tra le Alpi e il Bacino Mediterraneo.

[CR12] Cevasco R, Poggi G, Galante Garrone G, Griseri A, Lombardini S, Mamino L, Torre A (1999). Per una definizione storica del patrimonio rurale delle Valli Monregalesi: alpeggi della ‘raschera’. Le risorse culturali delle valli monregalesi e la loro storia.

[CR13] Chang C, Koster HA, Shiffer MB (1986). Beyond bones: toward an archaeology of pastoralism. Advances in archaeological method and theory, Volume 9.

[CR14] Chilès J-P, Delfiner P (1999). Geostatistics: modeling spatial uncertainty.

[CR15] Cliff AD, Ord JK (1973). Spatial autocorrelation.

[CR16] Crawley M (2007). The R book.

[CR17] Cribb R (1991). Nomads in archaeology.

[CR18] David N, Kramer C (2001). Ethnoarchaeology in action.

[CR19] Domínguez-Rodrigo M, de Juana S, Galàn AB, Rodríguez M (2009). A new protocol to differentiate trampling marks from butchery cut marks. Journal of Archaeological Science.

[CR20] Eve SJ, Crema ER (2014). A house with a view? Multi-model inference, visibility fields, and point process analysis of a Bronze Age settlement on Leskernick Hill (Cornwall, UK). Journal of Archaeological Science.

[CR21] Foster SM (1989). Analysis of spatial patterns in buildings (access analysis) as an insight into social structure: examples from the Scottish Atlantic Iron Age. Antiquity.

[CR22] Gifford DP, Gould RA (1978). Ethnoarchaeological observations of natural processes affecting cultural materials. Explorations in ethnoarchaeology.

[CR23] Gifford DP, Behrensmeyer AK (1977). Observed formation and burial of a recent human occupation site in Kenya. Quaternary International.

[CR24] Gifford-Gonzalez DP, Damrosch DB, Damrosch DR, Pryor J, Thunen RL (1985). The third dimension in site structure: an experiment in trampling and vertical dispersal. American Antiquity.

[CR25] Giraudoux, P. (2015). Package ‘pgirmess’: data analysis in ecology. R package version 1.5.9. http://cran.r-project.org/web/packages/pgirmess/index.html.

[CR26] Hillier B, Hanson J (1984). The social logic of space.

[CR27] Kvamme KL (1990). Spatial autocorrelation and the classic Maya collapse revisited: refined techniques and new conclusions. Journal of Archaeological Science.

[CR28] Legendre, P., & Legendre, L. (2012). *Numerical ecology, 3rd* (English ed.). Amsterdam: Elsevier Science BV.

[CR29] Lloyd CD, Atkinson PM (2004). Archaeology and geostatistics. Journal of Archaeological Science.

[CR30] Mackie C (2014). Crossing the threshold: negotiating space in the vernacular house of the Isle of Lewis. The Archaeological Journal.

[CR31] Mamino L (2001). Atlante dell’Edilizia Montana nelle Alte Valli del Cuneese. 1. Le Valli Monregalesi (Valli Casotto, Corsaglia, Maudagna, Ellero).

[CR32] Markofsky S, Bevan A (2012). Directional analysis of surface artefact distributions: a case study from the Murghab Delta, Turkmenistan. Journal of Archaeological Science.

[CR33] McBrearty S, Bishop L, Plummer T, Dewar R, Conard N (1998). Tools underfoot: human trampling as an agent of lithic artifact edge modification. American Antiquity.

[CR34] Moran PAP (1950). Notes on continuous stochastic phenomena. Biometrika.

[CR35] Neteler M, Mitasova H (2008). Open source GIS: a GRASS GIS approach.

[CR36] Nielsen AE (1991). Trampling the archaeological record: an experimental study. American Antiquity.

[CR37] O’Connell JF (1987). Alyawara site structure and its archaeological implications. American Antiquity.

[CR38] Ohser J (1983). On estimators for the reduced second moment measure of point processes. Mathematische Operationsforschung und Statistik, Series Statistics.

[CR39] Olsen SL, Shipman P (1988). Surface modification on bone: trampling versus butchery. Journal of Archaeological Science.

[CR40] Orton C (2004). Point pattern analysis revisited. Archeologia e Calcolatori.

[CR41] Palmisano, A. (2013). Zooming patterns among the scales: a statistics technique to detect spatial patterns among settlements. In G. Earl, T. Sly, A. Chrysanthi, P. Murrieta-Flores, C. Papadopoulos, I. Romanowska & D. Wheatley (Eds.), *Archaeology in the Digital Era. Papers from the 40th Annual Conference of Computer Applications and Quantitative Methods in Archaeology (CAA), Southampton, 26–29 March 2012*, (pp. 348–356). Amsterdam: Amsterdam University Press.

[CR42] Pebesma EJ (2004). Multivariable geostatistics in S: the gstat package. Computers & Geosciences.

[CR43] Premo LS (2004). Local spatial autocorrelation statistics quantify multi-scale patterns in distributional data: an example from the Maya Lowlands. Journal of Archaeological Science.

[CR44] Ripley BD (1976). The second-order analysis of stationary point process. Journal of Applied Probability.

[CR45] Roatta M, Roatta M, Galante Garrone G, Griseri A, Lombardini S, Mamino L, Torre A (1999). Gli edifici per l’abitazione e il lavoro. Tipologia, insediamenti, tecnologia e materiali. Le risorse culturali delle valli monregalesi e la loro storia.

[CR46] Rondelli B, Lancelotti C, Madella M, Pecci A, Balbo A, Ruiz Pérez J, Inserra F, Gadekar C, Cau Ontiveros MÁ, Ajithprasad P (2014). Anthropic activity markers and spatial variability: an ethnoarchaeological experiment in a domestic unit of Northern Gujarat (India). Journal of Archaeological Science.

[CR47] Rosso G (1950). Vita economica, insediamento stagionale, tipi di abitazione nelle valli superiori del Pesio e dell’Ellero nell’alto Monregalese. Atti dell’Accademia Ligure di Scienze e Lettere.

[CR48] Schiffer MB (1987). Formation processes of archaeological record.

[CR49] Shea JJ, Klenck JD (1993). An experimental investigation of the effects of trampling on the results of lithic microwear analysis. Journal of Archaeological Science.

[CR50] Simms SR (1988). The archaeological structure of a Bedouin camp. Journal of Archaeological Science.

[CR51] Stockton ED (1973). Shaw’s Creek Shelter: human displacement of artefacts and its significance. Mankind.

[CR52] Tobler, W. R. (1970). A computer movie simulating urban growth in the Detroit region. *Economic Geography, 46.* Supplement: Proceedings. International Geographical Union. Commission on Quantitative Methods, 234–240.

[CR53] Tomka SA, Cameron CM, Tomka SA (1993). Site abandonment behavior among transhumant agro-pastoralists: the effects of delayed curation on assemblage composition. Abandonment of settlements and regions: ethnoarchaeological and archaeological approaches.

[CR54] Vanzetti A, Vidale M, Gallinaro M, Frayer DW, Bondioli L (2010). The iceman as a burial. Antiquity.

[CR55] Villa P, Courtin J (1983). The interpretation of stratified sites: a view from underground. Journal of Archaeological Science.

[CR56] Wells EC (2010). Sampling design and inferential bias in archaeological soil chemistry. Journal of Archaeological Method and Theory.

[CR57] Yellen J (1977). Archaeological approaches to the present: models for reconstructing the past.

